# Birth Order and Family Size of UK Biobank Subjects Identified as Asexual, Bisexual, Heterosexual, or Homosexual According to Self-Reported Sexual Histories

**DOI:** 10.1007/s10508-024-03004-2

**Published:** 2024-10-01

**Authors:** Jan Kabátek, Ray Blanchard

**Affiliations:** 1 Melbourne Institute of Applied Economic and Social Research, The University of Melbourne, 111 Barry St., Carlton, Melbourne, VIC 3053 Australia; 2 ARC Centre of Excellence for Children and Families Over the Life Course, Brisbane, Australia; 3 Institute of Labor Economics, Bonn, Germany; 4 CentER, Tilburg University, Tilburg, The Netherlands; 5 Department of Psychiatry, The University of Toronto, Toronto, ON Canada

**Keywords:** Asexuality, Birth order, Bisexuality, Family size, Homosexuality, Maternal immune hypothesis

## Abstract

This study used a recently developed statistical technique to investigate the relations between various elements of a subject’s family background and the odds of that subject reporting a sexual history indicative of a minority sexual orientation. The subjects were 78,983 men and 92,150 women who completed relevant questionnaire items in the UK Biobank, a large-scale biomedical database of volunteers aged 40–69 years. The men were divided into three sexual minority groups—homosexual, bisexual, and asexual—and a comparison group of heterosexual men. The same was done for the women. The analytic procedure consisted of logistic regressions specifically designed to disentangle the effects of birth order and family size. The results showed that older brothers increased the odds of homosexuality in both men and women, and that older sisters increased the odds in men. In contrast, neither older brothers or older sisters affected the odds of bisexuality or asexuality in men or in women. These results suggest that birth order effects may be specific to homosexuality and not common to all minority orientations. The only family size finding was the negative association between family size and the odds of asexuality in both men and women. The outcomes of this study indicate that the maternal immune hypothesis, which was advanced to explain the relation between older brothers and homosexuality in later-born males, might have to be abandoned or else expanded to explain the findings concerning females. A few such modifications are considered.

## Introduction

Researchers have analyzed the family demographics of homosexual and heterosexual individuals for almost 90 years in the search for biological causes of homosexuality (Lang, 1936; Slater, 1962). This effort has so far yielded one reliable finding: Greater numbers of older brothers correlate with greater odds of homosexuality in later-born males. This repeatedly demonstrated phenomenon has been called the fraternal birth order effect (Blanchard & Klassen, 1997).

The analysis of other family relationships has produced less consistent results. Some studies have found that older sisters have no effect on the odds of homosexuality in later-born males (Blanchard & Bogaert, 1996a, 1996b; Blanchard et al., 1998; Ellis & Blanchard, 2001), some have found that older sisters have a weaker effect than older brothers (Ablaza et al., 2022; Blanchard & Lippa, 2021), and some have found that they have about the same effect as older brothers (Kangassalo et al., 2011; Schwartz et al., 2010; Semenyna et al., 2023). Another line of inquiry with inconsistent results is the relationship between birth order and sexual orientation in women. Some studies have found that older siblings have no effect on the odds of homosexuality in later-born females (Apostolou, 2020; Blanchard et al., 1998; Ellis & Blanchard, 2001), and some have found that older siblings have the same effect on females as on males (Ablaza et al., 2022; Fořt et al., 2024).

Researchers’ knowledge (or assumptions) about the roles, if any, that females play in the relationship between birth order and sexual orientation will naturally influence their theoretical accounts of this relationship. The best-known theory of the relationship, the maternal immune hypothesis (Blanchard, 2001)*,* was formulated at a time when the bulk of evidence suggested that older sisters have no effect on the odds of homosexuality in later-born males, and that neither older brothers nor older sisters have any effect on the odds of homosexuality in later-born females.

The maternal immune hypothesis proposes that cells (or cell fragments) from male fetuses enter the maternal circulation at some point during pregnancy. These cells include male-specific (i.e., Y-chromosome-linked) proteins that occur primarily on the surfaces of male brain cells. Maternal antibodies to these proteins cross the placental barrier, enter the fetal brain, and prevent its neurons from connecting in a fully male-typical pattern. This can result in a future homosexual orientation for a male fetus.

Recent research by Ablaza et al. (2022) has challenged the assumptions, crucial to the maternal immune hypothesis, of no birth order effect on females and no effect of females. Ablaza et al.’s sample of 9,073,496 male and female Dutch subjects (born between 1940 and 1990) was assembled using linked population registers maintained by the Dutch national statistics agency. These registers include data on same-sex marriage, which Ablaza et al. treated as a proxy for homosexuality. The authors found that older sisters do increase the odds of homosexuality in later-born males, although to a significantly lesser extent than do older brothers. They also found that the effects of older brothers and older sisters on females are similar to their effects on males. Besides the novelty of the presented findings, the study was also distinctive in using a new logistic regression model. The model enabled the authors to disentangle the influences of older siblings from the confounding influences of family size, thereby yielding more reliable estimates of the fraternal (and sororal) birth order effects.

It is important to replicate the study by Ablaza et al., not only because of the centrality of their findings to theory in this area, but also because they obtained these findings with a novel statistical technique that they developed specifically for their birth order analyses.^1^ Thus, the first goal of the present study was to conduct such a replication, using the UK Biobank, a large-scale biomedical database and research resource, which includes questionnaire data on approximately half a million UK participants.

Our second goal was to extend the findings for homosexuality to two other sexual minority groups, asexual and bisexual. The results for these groups, besides being interesting in themselves, might also bear on the interpretation of the fraternal birth order effect. Suppose, for example, that older brothers increase the odds of asexuality in later-born males just as they increase the odds of homosexuality in later-born males. This might suggest that the biological events triggered by older brothers tend to abolish sexual interest in women in addition to promoting sexual interest in men.

We may note here that the UK Biobank did not include any items asking subjects directly whether they are heterosexual, homosexual, bisexual, or asexual, using either those exact words or close synonyms. We do not believe that this prevents us from interpreting the results in terms of sexual orientation.

In our view, sexual orientation is a latent variable that cannot be observed directly using current technologies. Sexual orientation is necessarily inferred using one or more of three fallible indicators: (1) Self-reported erotic interests and fantasies, (2) enacted behavior, and (3) psychophysiological measurement.

In specific populations assessed under specific conditions, self-reported erotic interests may be reasonably assumed to be the least fallible indicator. That assumption would be different, however, from the position that sexual orientation is *defined* by self-reported erotic interests. Thus, we believe that the use of sexual histories to identify sexual orientation groups is not a matter of replacing the *true* measure of sexual orientation (self-reported erotic interests) with a clearly *untrue* substitute (sexual history). It is a matter of using one fallible indicator, which happens to be available in the UK Biobank, in lieu of another fallible indicator, which might have been less fallible but was not included in that database.

## Method

### Source Database

The UK Biobank is a large-scale biomedical database and research resource, which contains genetic and health information along with questionnaire data for 502,367 British participants aged 40–69 years at the time of recruitment.^2^ The recruitment started in 2006 and finished in 2010, with the data collection still ongoing at the time of writing. The questionnaire items used in our study were part of the baseline assessment that was administered upon recruitment. Some items were added to the assessment while the recruitment was already underway, and some have been discontinued, so that different items have different numbers of valid responses. Thus, the number of subjects who can be used in any given investigation is limited by the extent to which the required questionnaire items overlap. The number of respondents usable for the present study was 171,133 (Table 1). More detail on these subjects will be given after a description of the processes used to select them from the database and assign them to study groups.

**Table 1:** Numbers and percentages of male and female subjects in the four sexual orientation groups

Sex	Sexual orientation			
	Asexual	Bisexual	Homosexual	Heterosexual
Males	960 1%	710 1%	1369 2%	75,944 96%
Females	1033 1%	931 1%	416 < 1%	89,770 97%

### Materials and Procedure

#### Sexual Orientation

Four questionnaire items were used to assign subjects to one of four sexual orientation groups: asexual, bisexual, heterosexual, and homosexual. The first item was Data-Field 2139, “What was your age when you first had sexual intercourse? (Sexual intercourse includes vaginal, oral or anal intercourse).” Subjects whose responses indicated more than zero experiences of sexual intercourse were administered Data-Field 2149, “About how many sexual partners have you had in your lifetime?” The third item was Data-Field 2159, “Have you ever had sexual intercourse with someone of the same sex?” Subjects who indicated more than zero same-sex experiences were administered Data-Field 3669, “How many sexual partners of the same sex have you had in your lifetime?” It follows that subjects’ numbers of opposite-sex partners can be calculated by subtracting their numbers of same-sex partners from their total numbers of partners.

In order to classify respondents by sexual orientation, numbers of same-sex and opposite-sex partners were combined into one derived variable, Share of Same-Sex Partners, using the formula, same-sex partners ÷ (same-sex partners + opposite-sex partners). Respondents with a share value less than 0.20 were classified as heterosexual, respondents with a share value greater than or equal to 0.80 were classified as homosexual, and respondents with a share value between these two points were classified as bisexual.^3^ Respondents with zero lifetime same-sex and opposite-sex partners were classified as asexual.^4^

#### Sibship Composition

The sibship data needed for this research—the subject’s numbers of older brothers, older sisters, younger brothers, and younger sisters—were not included as standalone items in the UK Biobank questionnaire. These quantities therefore had to be computed from the sibship items described directly below, in conjunction with an algorithm that we designed to operate on these items.

Three questionnaire items were used in determining each respondent’s numbers of male and female, older and younger siblings. The first item was Data-Field 1873, “How many brothers do you have? (Please include those who have died, and twin brothers. Do not include half-brothers, step-brothers or adopted brothers).” The second item was Data-Field 1883, “How many sisters do you have? (Please include those who have died, and twin sisters. Do not include half-sisters, step-sisters or adopted sisters).” The third item was Data-Field 5057, “"How many OLDER brothers/sisters do you have? (Please include those who have died, and twins. Do not include half-, step- or adopted brothers and sisters).” A fourth datum, number of younger siblings, was computed as brothers + sisters − older siblings.

The item concerning older siblings was added to the questionnaire after the start of data collection, so this item had missing values for many subjects (this is the primary reason why our analytical sample is less than half the size of the full UK Biobank sample). The subjects with missing numbers of older siblings could not be used in the present research, with one exception: If the subject reported 0 brothers and 0 sisters, then the subject’s number of older siblings had to be 0.

**Fig. 1:**
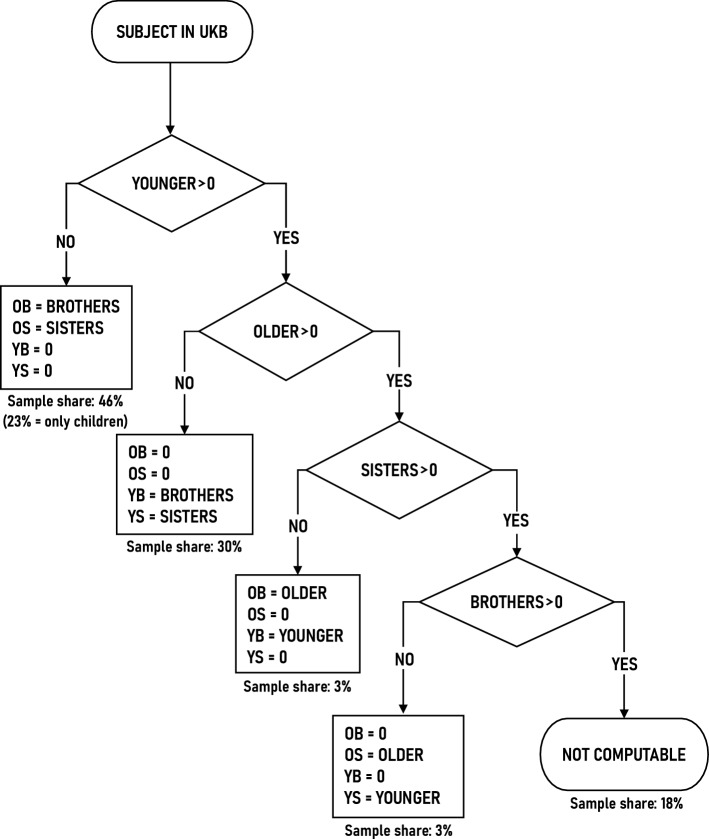
Calculation of sibship composition. Younger = number of younger siblings, older = number of older siblings, brothers = total number of brothers, sisters = total number of sisters, OB = older brothers, OS = older sisters, YB = younger brothers, YS = younger sisters. The “sample shares” are the percentages of subjects whose sibship composition (i.e., number of older brothers, older sisters, and so on) was computed by the corresponding path within the flow chart. The sample share data are given purely to help describe the subjects; sample share is not a variable that is used in any data analysis. The sample shares were computed on the subset of UK Biobank participants who responded to each of the sibling questions used in the assignment protocol, and who reported the number of their sexual partners

The flowchart presented in Fig. 1 represents the algorithm used to calculate the subjects’ numbers of older brothers, older sisters, younger brothers, and younger sisters. This algorithm begins by examining the subject’s number of younger siblings. If the subject reported no younger siblings, then any brothers that the subject reported must be older brothers and any sisters must be older sisters. If they reported that they did have younger siblings, then we proceeded further down the flowchart.

For some subjects, the numbers of older brothers, older sisters, and so on, were not computable. That is the outcome depicted in the lower right corner of the flow chart. This outcome corresponds to the subjects who are neither the oldest nor the youngest child in their sibship, and who have both brothers and sisters.

### Subject Characteristics

The numbers of male and female subjects in each sexual orientation group are shown in Table 1. The sample mean ages of the eight groups are given in Table 2. On average, heterosexual men and women were the oldest subjects, and the homosexual men and women were the youngest. Table 3 shows that, on average, the heterosexual men and women had the lowest educational attainment.

**Table 2:** Ages of the eight groups in years

Group	Estimated mean	Standard error	95% confidence interval
*Men*			
Heterosexual	57.12	0.03	57.07–57.18
Bisexual	54.99	0.3	54.40–55.59
Homosexual	53.19	0.22	52.76–53.61
Asexual	55.57	0.26	55.06–56.08
*Women*			
Heterosexual	56.43	0.03	56.38–56.49
Bisexual	52.47	0.26	51.95–52.98
Homosexual	51.95	0.39	51.19–52.72
Asexual	55.88	0.25	55.40–56.37

Mean values for the subjects in the respective sexual orientation groups are estimated using sample means. Numbers of observations are presented in Table 1

**Table 3:** Education attainment of the eight groups in years

Group	Estimated mean	Standard error	95% confidence interval
*Men*			
Heterosexual	13.28	0.01	13.26–13.30
Bisexual	14.03	0.12	13.79–14.26
Homosexual	14.24	0.09	14.07–14.41
Asexual	13.89	0.10	13.68–14.09
*Women*			
Heterosexual	13.24	0.01	13.22–13.26
Bisexual	14.52	0.10	14.32–14.72
Homosexual	13.97	0.16	13.66–14.28
Asexual	14.31	0.10	14.11–14.51

Mean values for the subjects in the respective sexual orientation groups are estimated using sample means. Numbers of observations are presented in Table 1

## Results

The sample mean numbers of siblings for the eight groups are presented in Table 4. On average, the asexual males and females had the lowest number of siblings.

**Table 4:** Estimated means and standard errors of relevant sibship characteristics

	Heterosexual (ref)	Bisexual	Homosexual	Asexual
*Men*				
Number of siblings	1.37	**1.49**	**1.49**	**1.11**
	(0.01)	(0.05)	(0.04)	(0.04)
Number of older siblings	0.59	**0.71**	**0.75**	**0.45**
	(0.00)	(0.04)	(0.03)	(0.03)
Number of younger siblings	0.79	0.79	0.74	**0.66**
	(0.00)	(0.05)	(0.03)	(0.04)
Number of older brothers	0.3	**0.36**	**0.39**	**0.25**
	(0.00)	(0.03)	(0.02)	(0.02)
Number of older sisters	0.28	**0.35**	**0.36**	**0.2**
	(0.00)	(0.02)	(0.02)	(0.02)
Number of younger brothers	0.41	0.43	**0.37**	**0.33**
	(0.00)	(0.03)	(0.02)	(0.03)
Number of younger sisters	0.37	0.36	0.37	**0.33**
	(0.00)	(0.03)	(0.02)	(0.02)
*Women*				
Number of siblings	1.39	1.44	1.42	**1.20**
	(0.00)	(0.05)	(0.07)	(0.04)
Number of older siblings	0.61	**0.69**	**0.75**	**0.51**
	(0.00)	(0.04)	(0.05)	(0.03)
Number of younger siblings	0.78	0.75	0.67	**0.69**
	(0.00)	(0.04)	(0.06)	(0.04)
Number of older brothers	0.32	**0.37**	**0.43**	**0.26**
	(0.00)	(0.02)	(0.03)	(0.02)
Number of older sisters	0.29	0.32	0.31	0.25
	(0.00)	(0.02)	(0.03)	(0.02)
Number of younger brothers	0.40	0.38	0.35	0.36
	(0.00)	(0.03)	(0.04)	(0.02)
Number of younger sisters	0.38	0.36	0.32	**0.33**
	(0.00)	(0.03)	(0.04)	(0.02)

Mean values for the subjects in the respective sexual orientation groups are estimated using sample means. Numbers of observations are presented in Table 1. Bold print indicates that the mean estimate is significantly different from the sample mean of the reference group (heterosexual respondents of the same gender) at 5% significance level

Table 5 shows the ratio of older brothers to older sisters for each of the groups. These ratios have been expressed as males:females × 100 and compared with the human sex ratio at birth, namely 106 males per 100 females (Chahnazarian, 1988; James, 1987). The most important findings are those for the heterosexual males and females, because these groups are meant to represent the general population (in comparisons involving the sexual minority groups). The results show that the ratio for heterosexual females is significantly skewed toward older brothers, which may signal that this group is not fully representative of the general female population.

**Table 5:** Ratio of older brothers to older sisters

Sex	Sexual orientation			
	Asexual	Bisexual	Homosexual	Heterosexual
Males	128	102	109	108
Females	107	115	137	**113**

Bold print indicates that the estimated sex ratio is significantly different from 106:100 at 1% significance level

The statistical relations between older brothers, older sisters, and family size and the odds that a subject will manifest a minority sexual orientation (asexuality, bisexuality, or homosexuality) were investigated in a series of binary logistic regression analyses. These multivariate regressions used the model introduced by Ablaza et al. (2022) and simplified by Blanchard (2022), following a suggestion offered by Ablaza et al. themselves.

The full procedure for any given comparison (for example, homosexual vs. heterosexual males) consisted of two logistic regression models. In both models, the criterion variable was a dichotomous dummy variable representing the subject’s sexual orientation—in this example, homosexual or heterosexual. The two analyses differed only in regard to their predictor variables, as explained below.

In the first model, the predictor variables were Number of Siblings, Number of Older Siblings, and Number of Older Brothers. This model will be referred to as the *baseline parameterization* (see Table 6). The regression coefficients produced by this model represent the effect of sibship size, the effect of older sisters, and the difference in magnitude between the effects of older brothers and older sisters, respectively.

**Table 6:** Parameterization of logistic regressions

Predictor variable	Coefficient	Interpretation of coefficient
*Baseline parameterization*		
Number of siblings	β_1_	One younger sibling is added to sibship
Number of older siblings	β_2_	One older sister replaces one younger sibling
Number of older brothers	β_3_	One older brother replaces one older sister
*Complementary parameterization*		
Number of siblings	δ_1_	One younger sibling is added to sibship
Number of older siblings	δ_2_	One older brother replaces one younger sibling
Number of older sisters	δ_3_	One older sister replaces one older brother

The second model was estimated for the sole purpose of retrieving the effect of older brothers. In this model, which is called the *complementary parametrization* (see Table 6), the predictor variable Number of Older Sisters replaced Number of Older Brothers. In this second model, the regression coefficient for Number of Older Siblings represents the effect of older brothers rather than the effect of older sisters.

Table 7 lists the four unique coefficients from the two regression models, along with their interpretations and the labels used in this article. We introduced the parameter labels (and acronyms) used in Table 7 for a few reasons. We used the labels older brother swap effect (OBSE) and older sister swap effect (OSSE) instead of fraternal birth order effect and sororal birth order effect, because the latter terms are becoming increasingly ambiguous. Different studies use these terms to label coefficients that have very different real-life interpretations, which creates a false sense of comparability and leads some commentators (and researchers alike) to misinterpret the state of the literature and its implications. We used OBSE and OSSE to emphasize our choice of quantification operations. The third parameter, the brother–sister swap effect (BSSE), may be thought of as the difference in magnitude between the fraternal and sororal birth order effects.

**Table 7:** Interpretation and labeling of regression coefficients

Model parameterization	Predictor	Coefficient	Interpretation	Full label	Acronym
Complementary	Number of older siblings	δ_2_	One older brother replaces one younger sibling	Older brother swap effect	OBSE
Baseline	Number of older siblings	β_2_	One older sister replaces one younger sibling	Older sister swap effect	OSSE
Baseline	Number of older brothers	β_3_	One older brother replaces one older sister	Brother–sister swap effect	BSSE
Baseline	Number of siblings	β_1_	One younger sibling is added to sibship	Younger sibling addition effect	YSAE

Another way of interpreting the regression coefficients, taking δ_2_ as an example, is as follows: δ_2_ estimates the difference in likelihood of sexual minority status between two groups that are identical in the size of their sibships but differ in the composition of their sibships. The first group has one more older brother than the second group, and the second group has one more younger sibling than the first group. For economy of expression, we say that the coefficient represents the effect of *replacing* a younger sibling with an older brother or *swapping* an older brother for a younger sibling

We also wanted to create a terminology that stays close to the data and minimizes surplus theoretical meaning. Thus, we used the term Younger Sibling Addition Effect (YSAE) instead of *female fecundity effect* (FF; Khovanova, 2020) or *antagonistic effect* (AE; Raymond et al., 2023), because the latter terms, especially *antagonistic effect*, employ a theoretical interpretation of the relevant finding as a description of the finding. Our term, YSAE, is roughly equivalent to the *effect of family size* (that is, adding one sibling to the participant’s sibship, without changing the birth order of the participant or the characteristics of participant’s older siblings)*.*

In Table 7, the links between the predictor variables and the interpretations of their regression coefficients may not be obvious. Why, for example, should the same predictor variable, Number of Older Siblings, be interpreted in terms of older brothers when Number of Older Sisters is a predictor variable, but be interpreted in terms of older sisters when Number of Older Brothers is a predictor variable? The answer is that these links are mediated by the *ceteris paribus* condition, a feature of multiple regression that is key to Ablaza et al.’s whole approach (Ablaza et al., 2022). The Latin phrase *ceteris paribus* means “other things being equal.” In the context of multiple regression, it means interpreting the effect of a one-unit change in a given predictor variable if all other predictor variables are kept constant.

Thus, in the baseline parameterization shown in Table 6, increasing the number of older siblings translates into increasing the number of older sisters, because the number of older brothers is a predictor that needs to be held constant. Similarly, an increase in the number of older siblings must be accompanied by a decrease in the number of younger siblings, because the total number of siblings is also held constant. By the same logic, in the complementary parameterization, increasing the number of older siblings translates into increasing the number of older brothers, and it must be accompanied by a decrease in the number of younger siblings. The corresponding parameter estimates are labeled “swap effects” in Table 7, because of the exchange-like nature of their interpretations.^5^

**Fig. 2:**
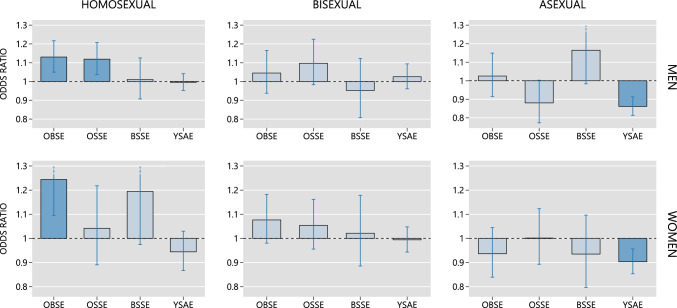
Predicted changes in the odds of a sexual minority preference associated with the explanatory variables of the *preferred* logistic regression model (see Table 7). Data sourced from the UK Biobank. OBSE (older brother swap effect) = effect of older brothers on the odds of a sexual minority orientation. OSSE (older sister swap effect) = effect of older sisters. BSSE (brother–sister swap effect) = difference in magnitude between the effect of older brothers and the effect of older sisters. YSAE (younger sibling addition effect) = effect of family size. Whiskers denote 95% robust confidence intervals. Estimates that are significantly different from the no-effect value (1.0) at 5% confidence level are denoted by shaded bars. To maintain consistent scaling across charts, the upper part of the confidence interval corresponding to BSSE on male asexuality has been truncated

The paired regression analyses were run a total of six times, in order to compare homosexual and heterosexual males, bisexual and heterosexual males, asexual and heterosexual males, homosexual and heterosexual females, bisexual and heterosexual females, and asexual and heterosexual females. The results of these analyses are depicted in Fig. 2.

The interpretation of this figure will be illustrated with the results for the homosexual males, which are shown in the upper left panel. The horizontal reference line at 1.0 represents no change in the odds of homosexuality. An odds ratio above 1.00 represents an increase in those odds, and an odds ratio below 1.0 represents a decrease in the odds.

The leftmost bar in this panel shows that adding one older brother to a sibship, while controlling the number of older sisters and the total number of siblings, significantly increased the odds of homosexuality. The next bar shows that adding one older sister to a sibship, while controlling the number of older brothers and the total number of siblings, also significantly increased the odds of homosexuality. The third bar from the left shows that there was no significant difference between the effect of older brothers and the effect of older sisters. The rightmost bar shows that increasing family size by one sibling while controlling for birth order had no effect on the odds of homosexuality.

**Fig. 3:**
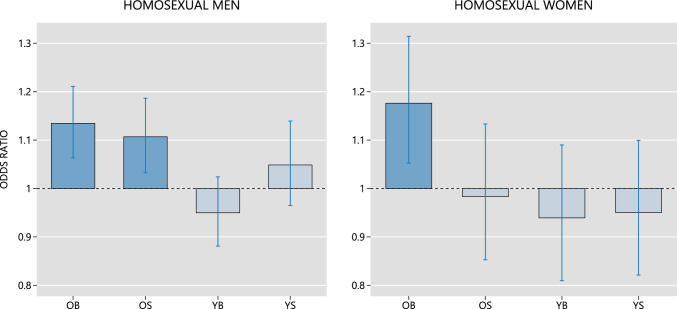
Predicted changes in the odds of homosexual preference associated with the explanatory variables of the *conventional* logistic regression model. Data sourced from the UK Biobank. OB (older brothers) = effect of adding an older brother to the sibship. OS (older sisters) = Effect of adding an older sister. YB (younger brothers) = Effect of adding a younger brother. YS (younger sisters) = Effect of adding a younger sister. Whiskers denote 95% robust confidence intervals. Estimates that are significantly different from the no-effect value (1.0) at 5% confidence level are denoted by shaded bars

We carried out two additional analyses, using the homosexual and heterosexual subjects only, to compare the foregoing logistic regression results with those obtained using the conventional regression model. In the conventional model (i.e., the model introduced by Blanchard & Bogaert, 1996b) the predictor variables are the subject’s number of older brothers, number of older sisters, number of younger brothers, and number of younger sisters. As can be seen in Fig. 3, the preferred and conventional models led to similar conclusions for both the male and female subjects.

## Discussion

One of the most striking findings of this study was the limitation of birth order effects to the homosexual groups. This suggests that birth order effects may be specific to homosexuality and not common to all minority orientations. In what follows, we discuss the homosexual groups first and then the sexual minority groups more generally.

**Fig. 4:**
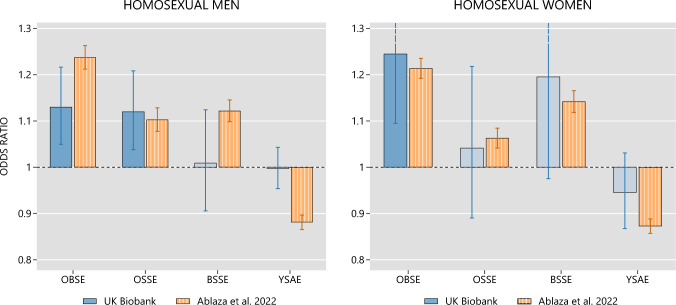
Comparison of the coefficient estimates corresponding to the logistic regression model of homosexual preference (UK Biobank sample) and the logistic regression model of same-sex union entry (Dutch population, as reported by Ablaza et al., 2022). Data sourced from the UK Biobank. OBSE (older brother swap effect) = effect of older brothers on the odds of a sexual minority orientation. OSSE (older sister swap effect) = effect of older sisters. BSSE (brother–sister swap effect) = difference in magnitude between the effect of older brothers and the effect of older sisters. YSAE (younger sibling addition effect) = effect of family size. Whiskers denote 95% robust confidence intervals. Estimates that prove significantly different from the no-effect value (1.0) at 5% confidence level are denoted by shaded bars

It is instructive to compare our findings on homosexuality with the findings from Ablaza et al.’s study, because that is by far the largest-ever study of family demographics and homosexuality, and because it used essentially the same statistical approach as the present study. The similarities and differences in results are presented graphically in Fig. 4. The results of these studies agreed completely with regard to the effects of older brothers. Both studies found that older brothers increase the odds of homosexuality in men and in women. There was somewhat less agreement with regard to the effects of older sisters. Both studies found that older sisters increase the odds of homosexuality in men, but only Ablaza et al. found that older sisters increase the odds of homosexuality in women. It might be noted that the former finding (older sisters increase the odds of homosexuality in men) argues against the possibility that Ablaza et al.’s OSSE findings merely reflected more supportive same-sex marriage attitudes among female relatives.

There was no confirmation of Ablaza et al.’s findings for family size. Ablaza et al. found that smaller family sizes were associated with higher odds of homosexuality in both sexes; the present study did not find any significant relation between family size and homosexuality in either sex.

Comparisons of the remaining coefficient, the brother–sister swap effect (BSSE), are limited by statistical power. The confidence interval corresponding to the UK Biobank sample overlaps with both the null effect and the confidence interval of Ablaza et al. (2022). This means that we cannot rule out the possibility of older brothers having no greater effect than older sisters or the opposite possibility of older brothers having a greater effect than older sisters, as documented in the Dutch population data.

There are various reasons to regard Ablaza et al.’s BSSE findings as more credible than the present BSSE findings. (1) The sample size in the Ablaza et al. study was an order of magnitude greater than the sample size in the present study. (2) A large amount of prior research has found that homosexual males have an excess of older brothers compared with their number of older sisters, whereas the same statistical methodology applied to heterosexual controls has found no excess of older brothers (e.g., Blanchard & Skorska, 2022, Figs. 2 and 3). (3) There are definite problems with the raw data on sibship composition in the UK Biobank, a matter discussed in greater detail below. It is likely that the statistical power for testing the BSSE is lower than the statistical power for testing the OBSE, OSSE, or YSAE. That is because the BSSE does not reflect the magnitude of an effect but rather the magnitude of a difference between two effects. Thus, noisy raw data could present a greater problem in identifying a BSSE.

The only family demographic effect we observed for the other two sexual minority groups (asexual and bisexual individuals) was the finding that asexuality was associated with smaller family sizes in both men and women. This finding disagrees with that of Yule et al. (2014), who found a fraternal birth order effect for asexual men. It also disagrees with the finding of Zdaniuk et al. (2024) that asexuality was associated with larger family sizes in men.

There are various ways that our result for the asexual subjects could have arisen. First, the mothers of asexual individuals might have been less fecund than average for purely physiological reasons. Second, the asexual groups, through some obscure recruitment bias, might have included an over-representation of subjects whose mothers had smaller ideal family sizes than the population average. A similar possibility is that the asexual groups, who essentially had zero fertility by definition, had mothers with lower fertility because of intergenerational transmission of notions of ideal family size (e.g., Anderton et al., 1987; Berent, 1953). The findings regarding asexuality and family size could, of course, have multiple reasons, including reasons that the present authors have not thought of. It would be premature to speculate further about these reasons until further research on asexual subjects has established the reproducibility of the findings.

Analyzing the data using the conventionally parameterized regression model (Blanchard & Bogaert, 1996b) led to similar conclusions for both the male and female subjects (cf. Figs. 2 and 3). We write *similar* rather than *identical* because there are subtle differences in the precise interpretations of the analogous parameters in the different models (see Ablaza et al., 2022).

### Limitations

As discussed in the Method section, the UK Biobank was not designed to study the relationship between family demographics and minority sexual orientations. Researchers who wish to use the database for that purpose must make the best use of items that happened to be included in its questionnaire. Such opportunistic use of the UK Biobank is directly related to two of the three study limitations that must be considered.

The first possible limitation concerns the assignment of subjects to sexual orientation groups. Survey researchers would ideally have multiple items for doing this, including several items on sexual behaviors and several items on sexual desires. We had only two items, both of which concerned sexual behaviors. It is most likely that the availability of more items would have increased reliability of classification, for the usual psychometric reasons that multi-item measures of psychological traits are preferable to single-item measures.^6^ We do not regard this as an especially serious problem, because alternative indices of sexual orientation will likely intercorrelate highly within samples of non-clinical research volunteers who have no special reason to lie about their sexual histories or interests. Thus, a large number of items is not essential for adequate reliability of sexuality classification, although two is certainly suboptimal. In any event, any limitation related to the possible misclassification of some subjects seems less serious than the limitation discussed next, which is specific to the UK Biobank.

The UK Biobank items concerning sibship composition were decidedly problematic for our purposes. As previously explained, the questionnaire item, “How many brothers do you have?” includes the instruction “Do not include half-brothers, step-brothers or adopted brothers.” Parallel instructions were given for reporting number of sisters and number of older siblings. There does not appear to be any way that respondents could report their numbers of half-siblings, which are not rare. Maternal half-siblings should be included in the counts—or at least available for analysis—if one is considering certain hypotheses, including the hypothesis that birth order effects reflect maternal–fetal immune interactions (e.g., Bogaert et al., 2018).

A review of the relevant demography literature (summarized in the Appendix) indicates that about 1 in 6 adults have or had a half-sibling. Two ad hoc analyses of archived data (also reported in the Appendix) suggest that the missing half-siblings would not be randomly scattered throughout the UK Biobank sample but rather concentrated among cases with no full siblings or relatively low educational levels. Thus, error in the family demographic data, even more than error in the sexual history data, may have affected the magnitude of birth order effects observed in this study.

The third and final issue that we will discuss concerns the heterosexual control groups. Blanchard and Skorska (2022) recommended that researchers undertaking a study of birth order and sexual orientation should run a preliminary test to see how well the heterosexual group represents the general population. These authors suggested computing the ratio of older brothers to older sisters collectively reported by a proposed control group and comparing that ratio with the expected value of 106 male live births per 100 female live births. They further suggested that, if the older sibling sex ratio of a proposed heterosexual control group differs “markedly and reliably” from the expected ratio of 106:100 (or proportion of 0.515), researchers might discard the entire sample, at least for purposes of studying sibship composition and sexual orientation.

Blanchard and Skorska did not offer any criterion for a “marked” departure from the expected value. The context of their remarks concerned a sample in which the older sibling sex ratio for both the heterosexual male and heterosexual female groups was 113. The ratios in the present study were 108 (n.s.) and 113 (*p* < .01) for heterosexual males and females, respectively. The present authors opted not to adopt Blanchard and Skorska’s draconian solution and discard the female subjects, but rather to note the potential for distortions in the write-up of the results.

In summary, the imperfect match between the available questionnaire items and the variables they were used to represent likely resulted in the presence of substantial noise in the data. This limits the confidence that one can place in the quantitative estimates of parameters such as the OBSE and the OSSE.

### Theoretical Implications

The foundational notions of the maternal immune hypothesis are that the effect of older siblings on later-born children takes place prenatally (see Bogaert, 2006) and that the maternal organ system best equipped to “remember” the number of fetuses a mother has previously carried is the immune system. The number of possible maternal–fetal immune interactions seems potentially large, especially when one considers that such interactions might influence fetal development and subsequent behavior without causing clinical problems or attracting research attention. Thus, the early findings that older sisters do not affect the sexual orientation of later-born males and that neither older brothers nor older sisters affect the sexual orientation of later-born females seemed to offer welcome constraints on the number of maternal–fetal immune reactions that one had to consider. Those constraints led to the hypothesis that the maternal–fetal immune interaction was triggered by male-specific (i.e., Y-chromosome-linked) antigen.

The more recent findings that older siblings of both sexes might influence sexual orientation in later-born children of both sexes do challenge the original version of the maternal immune hypothesis. These findings do not, however, touch the foundational notions underlying this hypothesis. It is therefore possible to start fresh and build an alternative hypothesis on the same foundations, a version that postulates antigens common to male and female fetuses. In what follows, we will briefly consider this possibility, along with the possibility of simply extending the existing version of the hypothesis. This discussion is somewhat complicated. We have therefore included Table 8 as a roadmap to it.

**Table 8:** Potential changes to original maternal immune hypothesis in response to recent findings

Older findings	Recent findings	Proposed mechanism for recent findings	Theoretical implications
Older brothers do not affect the odds of homosexuality in later-born females	Older brothers increase the odds of homosexuality in later-born females as well as in later-born males. (Ablaza et al., 2022; present study)	Epitope spreading	Original version of maternal immune hypothesis needs to be expanded but not abandoned
Older sisters do not affect the odds of homosexuality in later-born children	Older sisters increase the odds of homosexuality in later-born children, but less than older brothers. (Ablaza et al., 2022)	Correlation of live-born older sisters with miscarried male fetuses	Original version of maternal immune hypothesis requires no alteration
Older sisters do not affect the odds of homosexuality in later-born children	Older sisters increase the odds of homosexuality in later-born children as much as older brothers. (present study)	Autosomal antigen	Original version of maternal immune hypothesis must be replaced with alternative version that posits an autosomal rather than a sex-linked antigen

#### Effect of Older Brothers on Later-Born Females

The original version of the maternal immune hypothesis is attractive both because of its parsimony and because of its empirical support from the one relevant laboratory study (Bogaert et al., 2018). It is not difficult, at least conceptually, to defend its plausibility from one of the two incongruent findings, namely the finding that older brothers increase the odds of homosexuality in later-born females as well as in later-born males. The most detailed defense was formulated by Blanchard and Skorska (2022).

Blanchard and Skorska focused their discussion on NLGN4Y, a sex-dimorphic, Y-linked protein that is expressed in male fetal brain. Antibodies to NLGN4Y have been found at higher concentrations in sera from mothers of homosexual sons than in sera from mothers of heterosexual sons (Bogaert et al., 2018), thus supporting the speculation of Blanchard (2004) that NLGN4Y was a good candidate for the requirements of the maternal immune hypothesis. NLGN4Y has an X-linked homolog, NLGN4X, that is, an alternative form encoded by a gene on the X-chromosome. The two proteins are very similar, being about 97% identical in their amino acid sequences (Nguyen et al., 2020a, 2020b).

Blanchard and Skorska based their argument on the concept of *epitope spreading.* An *epitope* is a molecular region on the surface of an antigen that is capable of eliciting an immune response. A given antigen molecule may have multiple epitopes. *Epitope spreading* is a process whereby epitopes distinct from an inducing epitope subsequently become additional targets of an immune response.

Blanchard and Skorska argued that it is possible an antibody response initiated by epitopes found on NLGN4Y, but not on NLGN4X, subsequently spreads to epitopes on NLGN4Y that are also found on NLGN4X. Thus, it is conceivable that a maternal immune response initiated by a protein found only in male brain comes to target that protein as well as a similar protein found in female brain. There is no existing body of evidence from which one could specifically predict an effect of antibody-binding to NLGN4X on women’s sexual orientation. However, there is a role for NLGN4X in brain development in general, so it is plausible that its inactivation could increase the odds of future homosexuality in female fetuses.

The foregoing scenario is obviously speculative but not untestable. One could, for example, investigate whether the mothers of homosexual women have higher concentrations of antibody to NLGN4X/Y than do the mothers of heterosexual women. This would be an analogue of the study by Bogaert et al. (2018). In summary, the finding that older brothers increase the odds of homosexuality in later-born females as well as in later-born males—especially with further confirmation—would require extending the original version of the maternal immune hypothesis but not necessarily abandoning it.

#### Effect of Older Sisters on Later-Born Children

The other discrepant finding—that older sisters correlate with the odds of homosexuality in later-born children—presents a more complicated case. The implications for the maternal immune hypothesis depend on whether the statistical effect of older sisters is as strong as the effect of older brothers or significantly weaker. In the terminology used in this article, the implications depend on the absolute value and statistical significance of the BSSE.

The existence of a reliable but weaker sororal birth order effect would not only be compatible with the original maternal immune hypothesis; it was actually predicted by it. Blanchard and Lippa (2021) derived this prediction with the following reasoning: A mother’s number of live-born children will correlate with her number of early miscarriages. This correlation will be mediated by obvious factors such as maternal age. If live-born children correlate with miscarried fetuses, then number of live-born females will correlate with number of miscarried males. Miscarriages of male fetuses could expose mothers to significant quantities of fetal cells bearing Y-linked antigen (Khosrotehrani et al., 2003; Peterson et al., 2012). Thus, all other things being equal, mothers who have delivered more daughters are more likely to have “seen” male antigen than are mothers who have delivered fewer daughters. This implies the prediction of a weak correlation between a subject’s number of older sisters and that subject’s probability of being homosexual. Blanchard and Lippa’s prediction was confirmed in their study and subsequently by Ablaza et al. (2022) and Blanchard and Skorska (2022, Study 1). In conclusion, this finding, by itself, would not require any alteration of the original maternal immune hypothesis.

In contrast, the finding of equal size sororal and fraternal birth order effects, as in the present study, would be seemingly impossible for the original version of the maternal immune hypothesis to accommodate. In that case the most likely biological explanation for the finding of a high birth order for homosexual subjects would be an autosomal rather than a sex-linked antigen.

One would expect maternal–fetal immune interactions involving autosomal antigens, like those involving sex-linked antigens, to show birth order effects. In both cases, repeated pregnancies present the opportunity for repeated exposures to fetal antigen. This expectation is consistent with the epidemiology of certain fetal conditions caused by maternal–fetal blood group incompatibilities, most of which relate to autosomal antigens (e.g., Adams et al., 1981; Gualtieri et al., 1985; Petroff et al., 2022). Later-born infants are more likely to manifest these conditions.

Another line of research that is relevant to this point, although it does not focus on the health of the fetus, concerns the relation between maternal parity (number of completed pregnancies) and graft rejection in transplantation therapy. For example, the prevalence of antibodies to human leucocyte antigens (autosomal antigens) increases as a woman’s number of pregnancies increases. Such antibodies can lead to graft rejection (Alelign et al., 2018). In summary, proposing that an autosomal antigen causes the birth order effect in homosexuality appears just as feasible as proposing that a sex-linked antigen causes it.

The questions next arise whether maternal antibodies (or cytokines) stimulated by autosomal antigens could increase a fetus’s odds of future homosexuality, and if so, whether they could do this without producing conspicuous somatic anomalies. There are no empirical data that bear on these questions. It is therefore necessary to argue by analogy. Hall et al. (2023) recently reviewed the evidence that “maternal immune activation,” their term for a mother’s exposure to various immunogens during pregnancy, increases her offspring’s risk of neurodevelopmental disorders (e.g., autism spectrum disorder, attention-deficit/hyperactivity disorder, intellectual disability, communication disorder). Homosexuality is not a neurodevelopmental disorder; however, the two classes of phenomena seem comparable in that both are primarily mental/behavioral rather than somatic.

An autosomal antigen version of the maternal immune hypothesis would account for the newer findings (no difference between males and females) as parsimoniously as the Y-linked-antigen version of the hypothesis accounted for the older findings (only males are involved). There is, however, one finding that the alternative, autosomal hypothesis might seem unable to explain, namely higher concentrations of antibody to the Y-linked antigen NLGN4Y in mothers of gay men (Bogaert et al., 2018).

That finding is not necessarily an insuperable obstacle. The autosomal hypothesis could be reconciled with that finding via the auxiliary hypothesis that pregnant mothers of homosexual individuals are exposed to unusually high quantities of fetal cells or cell fragments, perhaps because some flaw in the placental interface allows such material to pass into the maternal compartment (Bianchi et al., 2021). According to this hypothesis, the pregnant mothers become exposed to some autosomal antigen *in addition to* NLGN4Y, and it is this autosomal antigen—not NLGN4Y—that triggers the maternal reaction leading to future homosexuality in the fetus. On this view, the elevated levels of anti-NLGN4Y antibody observed by Bogaert et al. would simply mean that the mothers of homosexual men were likely exposed to multiple fetal antigens, not that NLGN4Y itself was the antigen leading to future homosexuality in the fetus. Following this interpretation of Bogaert et al.’s results, the serum concentration of anti-NLGN4Y was simply a lucky choice of proxies for fetal–maternal cell traffic.

## Appendix

As previously stated, there does not appear to be any way for UK Biobank respondents to report their numbers of half-brothers or half-sisters. We therefore consulted the demography literature to guesstimate the proportion of UK Biobank cases whose sibship data were likely to be inaccurate because of unreported half-siblings. Next, we analyzed archived datasets in the possession of the second author (RB) to investigate whether that error would likely be randomly or systematically distributed among the cases. The best sources of information for these two questions concern American and Canadian subjects, but these are likely comparable to UK subjects, given the general similarity of the national cultures.

According to Knop (2020), who based his estimate on U.S. Census Bureau data, 1 in 6 American children live with a half-sibling under age 18. Monte (2017), who used the same database as Knop, found that 1 of 10 adults aged 15 or older had children with more than one partner. She reported that 1 in 6 parents had children with more than one partner, which resembles Knop’s finding. Guzzo tabulated data from several surveys (Guzzo, 2014, Table 1). Her calculation of the proportion of fathers with children by more than one woman, based on the dataset she described as the best source of U.S. fertility data, was about 1 in 6 for all fathers aged 40–44 and over 1 in 5 for fathers of this age with two or more children. She observed that the proportion of parents who had children with more than one partner is higher for persons with lower socioeconomic status and lower levels of education. Although none of the foregoing studies posed the exact question, “How many adults selected from the general population without regard to sibship composition have or had a half-sibling,” their study questions were similar enough that one can assume the answer is around the order of 1 in 6.

We used two archived samples with high quality family data in order to investigate whether the distribution of inaccurate/incomplete cases in the UK Biobank would likely be random or systematic. The first sample were the 2278 Canadian male subjects (mostly patients with sexual offenses) originally studied by Blanchard et al. (2012). Of that total, 164 responded “No” to the questionnaire item, “Have you had sufficient contact with your biological mother to be reasonably certain how many other children she gave birth to?” Those cases were not studied further. A substantial proportion of the remaining 2,114 men (19.9% or about 1 in 5) reported having one or more maternal half-siblings.

There was a significant negative correlation between the respondent’s number of full siblings and his number of maternal half-siblings, Spearman’s rho =  − 0.384, *p* < .001. By far, the largest mean number of maternal half-siblings, 1.60, was reported by subjects who reported zero full siblings. This result suggests that the most inaccurate family data in the UK Biobank likely concern the (seeming) only-children.

Subjects indicated their educational level on a questionnaire item that offered response options ranging from “less than Grade 8” to “graduate or professional degree.” The median education was high school graduation. There was a significant negative correlation, Spearman’s rho =  − 0.145, *p* < .001, between a subject’s educational level and his number of maternal half-siblings, possibly reflecting a social class difference in family stability.

The second sample were 877 primarily middle-class Canadian volunteers recruited for Blanchard and Bogaert (1996b). These included 141 men never analyzed for that study because of various elimination criteria (which included having any maternal half-siblings). These subjects were administered the same questionnaire later modified slightly for Blanchard et al. (2012).

There were 21 subjects who indicated uncertainty about their biological mother’s reproductive output and were therefore dropped from further analysis. Of the remaining 856 men, only 4.9% (about 1 in 20) reported having one or more maternal half-siblings. There was again, however, a significant negative correlation between the respondent’s number of full siblings and his number of maternal half-siblings, Spearman’s rho =  − 0.169, *p* < .001. In this sample, subjects with zero full siblings reported a mean of 0.39 maternal half-siblings.

In the Blanchard and Bogaert sample, the median education was university. There was no correlation between a subject’s educational level and his number of maternal half-siblings, Spearman’s rho =  − 0.025, n.s. This might reflect a lack of variability among these cases, 88.3% of whom had university or higher postsecondary education.

A simple Gedanken experiment illustrates why a negative correlation between full siblings and maternal half-siblings is predictable. Consider a hypothetical population in which all mothers have exactly three children by either one or two fathers. A man drawn at random from this population (Subject A) reports that he has two full siblings. He must, therefore, have zero half-siblings. Another man (Subject B) reports that he has no full siblings. He must, therefore, have two half-siblings.

Of course, mothers in real populations do not all have the same number of offspring. However, societal norms about ideal family size (which translate into stopping rules) will tend to move real populations in the direction of similar family sizes and thus toward the hypothetical postulated in the Gedanken experiment.

In summary, the UK Biobank dataset likely contains many cases whose sibship information is incomplete. The UK Biobank cases most likely to be inaccurate because of unreported/unreportable maternal half-siblings are those cases with no full siblings and relatively low educational levels.

## Footnotes

1 It is also important to assess the validity of same-sex marriage as a proxy for homosexuality, given that the decision to enter marriage may be subject to unrelated sibling influences. For example, women (including female relatives) appear more supportive of same-sex marriage than men (Baunach, 2012), which could lead to positive association between the number of older sisters and same-sex marriage (even in the absence of an association between the number of older sisters and sexual orientation).

2 https://www.ukbiobank.ac.uk/

3 The two threshold values were chosen following a visual inspection of the frequency of share values in our sample. We chose the threshold values that would yield relative frequencies of heterosexual, bisexual, and homosexual subjects that would be similar to the relative frequencies found in previous epidemiological research: that is, more homosexual than bisexual subjects for the males, and more bisexual than homosexual subjects for the females. The threshold values identified by this relatively informal method were intuitively satisfactory. It seems reasonable to label anyone with 80% or more same-sex partners as homosexual, and it seems reasonable to label anyone with 20–80% same-sex partners as bisexual. We note that changing the threshold values did not lead to substantive changes of our findings or their interpretation.

4 Some readers might object to the classification of respondents with zero lifetime sexual partners as asexual on the grounds that asexuality is a lack of sexual desire, not a lack of sexual experience. Given that we cannot observe the former, our labeling of the asexual group follows the same strategy as the labeling of the homosexual, heterosexual, and bisexual groups on the basis of sexual experience.

5 After we had completed the present study, Zdaniuk et al. (2024) reorganized the two logistic regression equations in a way that facilitates the exposition further. In their version, the first equation uses the predictors all siblings, older brothers, and older sisters; these predictors yield the estimates for YSAE, OBSE, and OSSE, respectively. The second equation uses the predictors all siblings, older siblings, and older brothers; the predictor older brothers yields the estimate for the BSSE. This arrangement is somewhat more intuitive because the predictor older brothers in the first equation estimates the effect of older brothers (OBSE) and the predictor older sisters in the first equation estimates the effect of older sisters (OSSE). Furthermore, the difference between the regression coefficients for older brothers and older sisters in the first equation is readily seen to equal the regression coefficient that directly estimates the brother–sister difference in the second equation (BSSE).

6 Some researchers might take the position that the only basis on which one can validly classify subjects as heterosexual, homosexual, and so on, is their self-report that such is their identity. As we have already indicated, we disagree with that position on epistemological and psychometric grounds. We believe that sexual orientation is a latent variable, and that the issues involved in its measurement are essentially the same psychometric issues involved in the measurement of any mental (as opposed to physiological) trait. In our view, the main shortcoming of our classification of sexual orientations is not that it is behavioral but that it is *only* behavioral.
